# Data on the respiratory sensor performance prepared with the MWCNT coated textile fabricated by vacuum-filtration

**DOI:** 10.1016/j.dib.2018.05.087

**Published:** 2018-05-23

**Authors:** Jaehwan Ko, Seunghyun Jee, Joo Hyeon Lee, Sun Hee Kim

**Affiliations:** a Department of Chemical and Biological Engineering, Gachon University, Seongnam, Republic of Korea; b Mcell Co., Ltd., Pangyo-ro 289beon-gil, Bundang-gu, Seongnam-si, Republic of Korea; c Department of Clothing and Textiles, Yonsei University, Seoul, Republic of Korea; d Department of Fashion Industry, Incheon University, Incheon, Republic of Korea

## Abstract

This data provides the respiratory sensor performance prepared with the MWCNT coated textile fabricated by vacuum-filtration. Readers are requested to go through the article entitled “High durability conductive textile using MWCNT for motion sensing” (Ko et al., 2018) [1] for further interpretation and discussion.

**Specifications Table**

**Table:** 

Subject area	*Physics*
More specific subject area	*Respiratory sensor using electrical resistance displacement*
Type of data	*Table, image, figure*
How data was acquired	*Keithley 2400 and Keithley 2000 source meter*
Data format	*Analyzed*
Experimental factors	*Respiratory sensor was prepared with MWCNT coated textile.*
Experimental features	*Respiratory sensor performance*
Data source location	*Energy Materials Lab, Department of Chemical Engineering, Gachon University, Seongnam, Korea.*
Data accessibility	*This article*

**Value of the data**

• Performance data on MWCNT coated textile based respiratory sensor.
• Comparison with commercial respiratory sensor.
• Conductive textile fabricated by vacuum-filtration of water-based MWCNTs ink.

## 1 Data

**Fig. 1:**
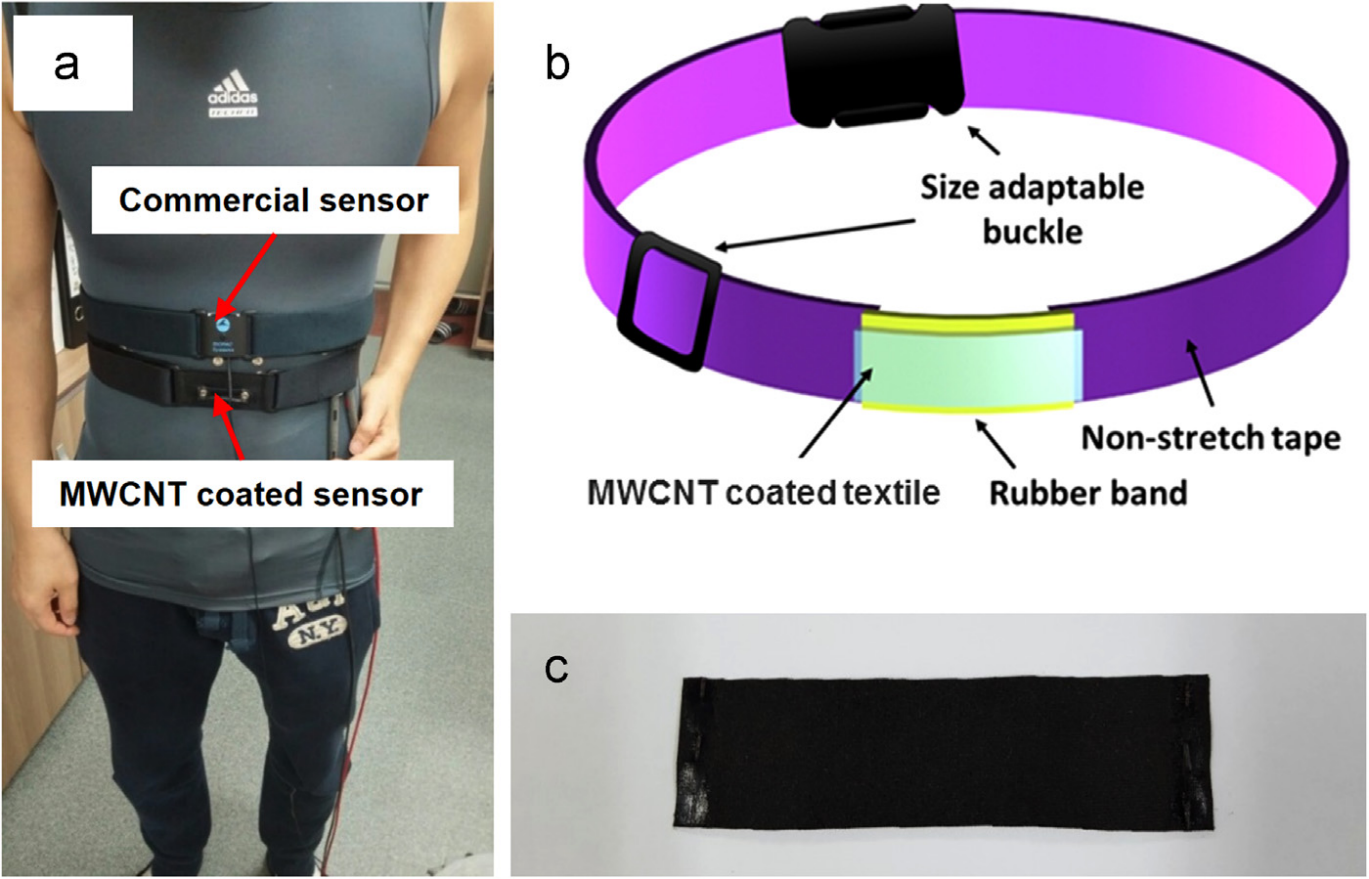
(a) Photograph of commercial respiratory sensor and the MWCNT coated textile based respiratory sensor; wearing at the upper abdomen, (b) Schematic of the MWCNT coated textile based respiratory sensor and (c) Photograph of MWCNT coated textile fabricated by vacuum-filtration.

**Fig. 2:**
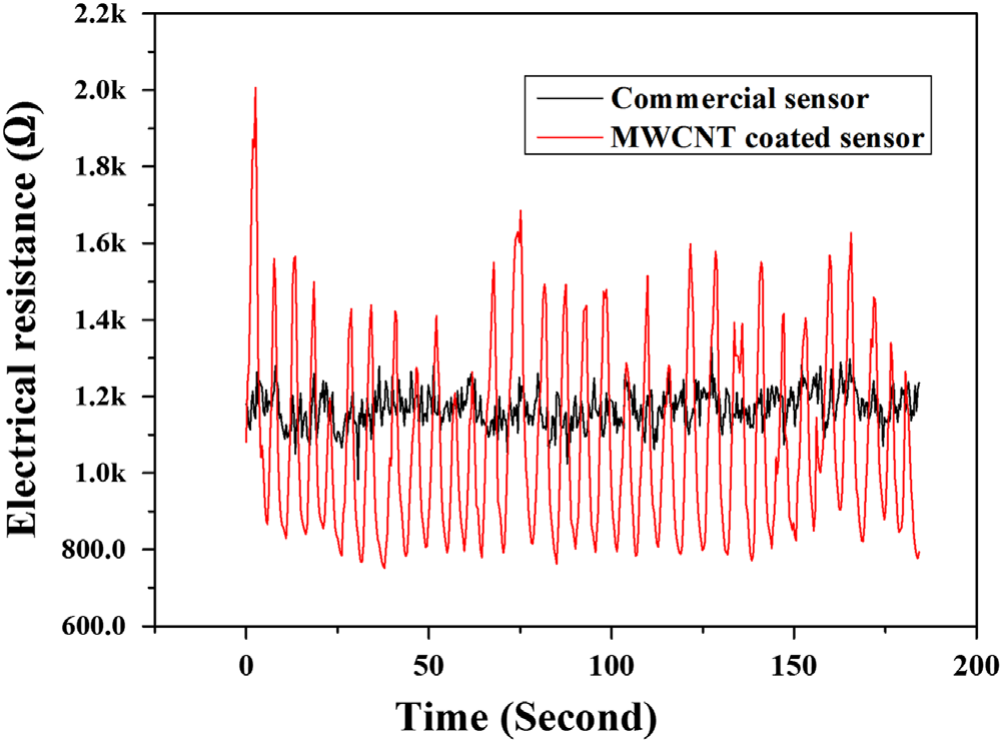
Variations in the electrical resistance as functions of respiration rates of commercial respiratory sensor and the MWCNT coated textile based respiratory sensor.

This dataset provides information on the performance of the respiratory sensor prepared with a conductive textile fabricated by vacuum-filtration of water-based MWCNTs ink. Fig. 1 shows the schematic of fabricated respiratory sensor and the wearing position of the sensor. Fig. 2 shows the variations in electrical resistance as functions of respiration rates of commercial sensor and fabricated sensor. The characteristic data like respiration per minute and sensitivity of the sensors are tabulated in Table 1.

**Table 1:** Results of respiration per minute and sensitivity of commercial respiratory sensor and the MWCNT coated textile based respiratory sensor.

**Sample**	**Respiration per minute**	**Sensitivity**
Commercial sensor	15.62	1.18
MWCNT coated sensor	15.84	1.79

## 2 Experimental design, materials and methods

A conductive textile was prepared by vacuum-filtration of water-based MWCNTs ink [1]. The MWCNT was oxidized in order to increase the distribution of the MWCNT particles in deionized (DI) water. Oxidation of the MWCNT was carried out by mixing 1 g of MWCNT in 200 ml of HNO_2_ in a three-neck flask. The flask was connected to a reflux-cooling device, and the contents were stirred on a heating mantle for 8 h at 150 rpm and 120 °C. The oxidized MWCNTs were neutralized until a final pH of 5. Next, 500 mg of oxidized MWCNTs were added to 500 mL of DI water and were dispersed using a sonicator. The sonication was carried out for 20 min at a power of 400 W and a frequency of 20 kHz. The textile used to prepare the conductive textile consisted of 93% polyester and 7% polyurethane. The MWCNTs ink was coated on the conductive threads stitched textile using the vacuum-filtration process. After vacuum-filtration, the wetted textile was dried at 70 °C for 1 h.

A respiration sensor was fabricated by installing a snap that imposes the conductive thread on the dried MWCNT coated conductive textile cut into dimensions of 4 cm × 1.5 cm. In order to evaluate the performance of the manufactured respiration sensor based on the MWCNT coated textile, the respiratory signals were compared with those of the commercial sensor. Each subject wore both the commercial sensor and the fabricated sensor at the upper abdomen, and the respiration patterns and the rates were measured simultaneously with the two sensors for 180 s at normal conditions while standing at ease, as shown in Fig. 1. The respiration rate was calculated as the number of breath cycles per 60 s.
